# Hemothorax after fetal pleural effusion-thoracoamniotic shunting procedure due to transient myeloproliferative disorder

**DOI:** 10.1515/crpm-2021-0032

**Published:** 2022-06-11

**Authors:** Yuriko Iwahata, Hideyuki Iwahata, Junichi Hasegawa, Chika Homma, Yoko Nishimura, Haruhiro Kondo, Nao Suzuki

**Affiliations:** Department Obstetrics and Gynecology, St. Marianna University School of Medicine, Kawasaki, Japan

**Keywords:** fetal pleural effusion, fetal pleural effusion-amniotic shunting, hemothorax, hydrops fetalis, thoracoamniotic shunting, transient myeloproliferative disorder, trisomy 21

## Abstract

**Objectives:**

To present a case of fetal hemothorax after two times of thoracoamniotic shunting (TAS) performed to pleural effusion with hydrops fetalis, resulting in non-reassuring fetal status due to hemothorax.

**Case presentation:**

This is a case of bilateral pleural effusion with hydrops fetalis and polyhydramnios at 32 weeks gestation, in which unilateral fetal TAS was performed twice, resulting in non-reassuring fetal status due to hemothorax. After delivery, the infant was diagnosed with trisomy 21 and transient myeloproliferative disorder (TMD) with disseminated intravascular coagulation and congenital systemic lymphangiopathy.

**Conclusions:**

In conclusion, since TAM case do not always show hemothrax, TAM is not inhibited but technical carefulness should be necessary.

## Introduction

Chylothorax, which is the most common form of primary pleural effusion in the prenatal period, occurs in approximately 1 in 10,000–15,000 pregnancies, with an overall mortality rate of 25% to 50% [1]. To avoid lung hypoplasia, heart failure, and hydrops fetalis due to compression by pleural effusion, fetal pleural effusion removal using thoracoamniotic shunting (TAS) are considered. Although the efficacy of TAS for fetal pleural effusion is well-recognized, complications may occur due to double-basket catheter insertion, such as the catheter tube getting lodged in the chest wall, hemorrhage from the chest wall or lung, infection, and preterm rupture of the membrane [2]. Herein, we present a case of bilateral pleural effusion with hydrops fetalis and polyhydramnios, in which unilateral fetal TAS was performed twice, resulting in non-reassuring fetal status due to hemothorax.

## Case presentation

**Figure 1:**
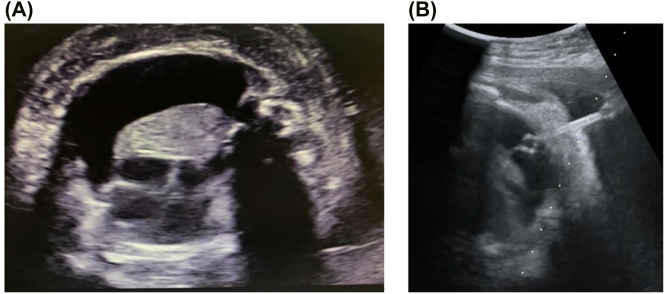
TAS insertion to fetal pleural effusion. (A) Right-dominant fetal pleural effusion. (B) During the TAS insertion, a shunt tube was placed in the fetal chest wall.

A 37 years-old nulliparous pregnant woman was referred to our perinatal center for fetal pleural effusion at 32 weeks of gestation. Fetal ultrasonography revealed right-dominant bilateral pleural effusion (Figure 1A), lung exclusion in the mediastinum, fetal subcutaneous edema, and hydrops fetalis. Fetal hepatomegaly (crossing the midline and occupying the right hypochondrium) was marked, and amniotic fluid index of 28 cm and polyhydramnios were observed; however, no other obvious morphological abnormalities were detected. The Doppler ultrasound findings included an umbilical artery resistance index (RI) of 0.77, a mid-cerebral artery (MCA) RI of 0.87, and an MCA peak systolic velocity of 36.6 cm/s. Blood tests were negative for cytomegalovirus, toxoplasmosis, rubella, and parvovirus B19.

Since pleural effusion resulted in increased intrathoracic pressure leading to polyhydramnios, fetal pleural effusion removal was attempted. Although we did genetic counseling to explain one of the causes of hydrops fetalis is chromosomal abnormalities, the patient and family did not want chromosome analysis. As the removed fluid (35 mL) was clear and light yellow, and had 92% of lymphocytes, a diagnosis of primary pleural effusion was established. Re-accumulation of pleural effusion was observed on the next day; thus, we performed TAS for the first time, and inserted a shunt tube through the right front chest wall of the fetus at 32+6 weeks of gestation. However, the tube slipped into the uterus on the following day, and the pleural effusion increased further. Two options, reinsertion of the shunt or follow-up and consideration of the delivery timing, were discussed. Finally, it was decided to attempt reinsertion of the shunt tube. At 33+1 weeks of gestation, the second TAS was performed, placement of the shunt tube in the precordium of the fetus was confirmed (Figure 1B), and the pleural effusion drained into the amniotic fluid cavity, as observed by color Doppler.

Nevertheless, 6 h after the procedure, cardiotocography (CTG) showed bradycardia, which decreased to a minimum of 40 bpm, and the infant was delivered by an extremely rapid emergency cesarean section. The birth weight was 2,415 g with an Apgar score of 1/1 (1/5 min), and umbilical artery pH of 6.87; the infant was intubated 4 min after birth. Even after intubation, the thoracic wall was hardly elevated. The shunt tube was inserted into the right chest, but there was no outflow of pleural effusion. Liquid retention was observed in the right thoracic cavity on ultrasound, and 117 mL of bloody pleural effusion was suctioned from the right thoracic cavity using a 17G needle.

After this procedure, an increase in heart rate and oxygen saturation was observed, resulting in obstructive shock due to right hemothorax. Blood sampling at birth showed an increase in leukocytes up to 69,700/μL, with a disseminated intravascular coagulation (DIC) score of 6 points. A diagnosis of transient myeloproliferative disorder (TMD) was established.

Although TMD improved spontaneously, chromosome analysis revealed trisomy 21. On day 30 after birth, anasarca continued, and bilateral pleural effusion and ascites were observed with continuous drainage. The infant was diagnosed with congenital systemic lymphangiopathy, and lymphatic anastomosis was performed on day 62 after birth. The anasarca improved in the short term but soon reappeared. On the 10th day after the operation, the general condition of the infant deteriorated owing to cellulitis of the left lower extremity, and her death was confirmed on the 98th day after the operation.

## Discussion

In our case, TAS was performed when the fetus had transient abnormal myelopoiesis (TAM), resulting in hemothorax and non-reassuring fetal status. From our perspective, hemothorax and DIC was occurred due to technical adverse events of TAS in TAM status and 21 trisomy case.

To establish a diagnosis of neonatal TAM without cordocentesis is quite difficult. According to previous reports, patients with trisomy 21 with TMD also present with hepatomegaly/hepatosplenomegaly during the fetal period [3, 4]. It has been reported that fetal hepatosplenomegaly and/or hydrops fetalis in the second half of pregnancy may be a sign of trisomy 21 and TMD [5]. Based on these reports and our case, ultrasonographic diagnosis of hepatosplenomegaly may be an important clue in predicting TMD and trisomy 21 in the antenatal period.

The prognosis of patients with hydrops fetalis is 21–35%, which is extremely low [6, 7]. Neonatal (or even fetal) death occurs in approximately 10% of patients secondary to diffuse infiltration. In other words, it is presumed that the prognosis was poor, regardless of the complications of administration of TAS, due to trisomy 21 and congenital systemic lymphangiopathy.

In conclusion, since TAM case do not always show hemothrax, TAM is not inhibited but technical carefulness should be necessary.
